# Novel Serotype of Epizootic Hemorrhagic Disease Virus, China

**DOI:** 10.3201/eid2612.191301

**Published:** 2020-12

**Authors:** Heng Yang, Zhuoran Li, Jinping Wang, Zhanhong Li, Zhenxing Yang, Defang Liao, Jianbo Zhu, Huachun Li

**Affiliations:** Yunnan Animal Science and Veterinary Institute, Kunming, China

**Keywords:** epizootic hemorrhagic disease virus, viruses, novel serotype, cattle, China

## Abstract

In 2018, a strain of epizootic hemorrhagic disease virus (EHDV), named YNDH/V079/2018, was isolated from a sentinel calf in Mangshi County, Yunnan Province, China. Nucleotide sequencing and neutralization tests indicated that the virus belongs to a novel serotype of EHDV that had not been reported previously.

Epizootic hemorrhagic disease virus (EHDV; family *Reoviridae*, genus *Orbivirus*) is transmitted between ruminants by *Culicoides* spp. biting midges. It is widespread in tropical and subtropical regions and primarily infects white-tailed deer and cattle (*1*); EHDV infection often causes death in white-tailed deer. Seven serotypes of EHDV (EHDV-1, -2, -4, -5, -6, -7, and -8) have been officially assigned; EHDV-3 (NIG1967/01 strain) has been combined into EHDV-1 (*1*,*2*). Recently, 2 novel EHDV strains isolated from South Africa (*3*) and Japan (*4*) were suggested as new serotype candidates. Although only Ibaraki virus (a strain of EHDV-2 from Japan) was previously known to cause a bluetongue-like illness in cattle (*1*), EHDV-1, EHDV-2, EHDV-6, and EHDV-7 have recently been associated with illness and death in cattle in Asia, the Mediterranean Basin, South Africa, North America, and Reunion Island (*1*,*5*–*7*), suggesting that the distribution and the pathogenicity associated with EHDV infection are increasing. EHDVs belonging to serotypes 1, 5, 6, and 7, as well as a nontyped serotype, have been isolated from sentinel cattle in southern China (H. Li et al., unpub. data).

In 2018, we placed 10 Yunnan yellow cattle 6−12 months of age and free of EHDV antibodies under field conditions at Sanjiaoyan village, Mangshi County, Dehong Prefecture, Yunnan Province, China (Appendix Figure 1) as sentinel animals. We took blood samples at weekly intervals during May−October: whole blood for serology, EDTA samples for viral nucleic acid detection, and heparin blood samples for virus isolation.

During June−September, we confirmed EHDV infections in 3 of the sentinel cattle by EHDV competitive ELISA (cELISA; ID-Vet, https://www.id-vet.com) and real-time quantitative reverse transcription PCR (qRT-PCR) (*3*). We isolated viruses from blood samples by inoculating C6/36 cells and blindly passaging for 5 times on BHK-21 cells (*1*). We isolated EHDV-1 and EHDV-5 strains from 2 of the cattle and an additional strain of EHDV, YNDH/V079/2018, from the third animal. Serotype identification of YNDH/V079/2018 displayed uniform negative results through serotype-specific RT-PCR (*8*) and virus neutralization tests using serum samples against EHDV-1, EHDV-2, EHDV-5, EHDV-6, EHDV-7, EHDV-8, and nontyped serotype reference strains. Furthermore, serum from the YNDH/V079/2018-infected calf showed no neutralization to tested serotypes of EHDV reference strains.

Double-stranded RNA extracted from YNDH/V079/2018 generated a genome segment migration pattern typical of bluetongue virus or EHDV (Appendix Figure 2) by agarose gel electrophoresis. Transmission electron microscopy revealed virus particles »80 nm in diameter, with a ring-shaped capsomere characteristic of *Orbivirus* (Appendix Figure 3). Full-length cDNA copies of segments 2 and 3 (Seg-2 and Seg-3) of YNDH/V079/2018 were synthesized and sequenced as described by Maan et al. (*9*). BLAST analyses (https://www.ncbi.nlm.nih.gov/BLAST) of Seg-2 and Seg-3 sequences (GenBank accession nos. MN418446 and MN418447) revealed the highest matching identities with equivalent genome segments of other EHDVs.

Subcore shell viral protein (VP) 3 of EHDV, encoded by Seg-3, is highly conserved, showing >95.5% amino acid sequence identity within EHDVs (*10*). Seg-3/VP3 of YNDH/V079/2018 showed overall nucleotide/amino acid identity levels of 78.5%/94.6% to 80.0%/96.5% with other EHDVs, confirming its identity as an EHDV isolate. However, Seg-3 of YNDH/V079/2018 did not cluster with previously identified Eastern or Western EHDV topotypes (*10*); maximum sequence identities were 80.0% nt and 78.9% aa, which placed YNDH/V079/2018 as a distinct topotype in the phylogenetic tree (Figure, panel A).

**Figure:**
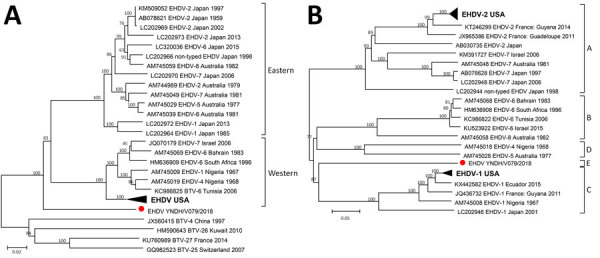
Phylogenetic analyses of EHDV based on segment 3 (A) and segment 2 (B) of YNDH/V079/2018 from Mangshi County, Yunnan Province, China (red dot), compared with other global EHDV isolates. The following convention was used to identify sequences: GenBank accession no., EHDV-serotype, country, isolation year. Eastern and Western topotypes of segment 3 and A−D groups of segment 2 were assigned as described by Anthony et al. (*2*,*10*); a distinct segment 2 group of the strain YNDH/V079/2018 isolated in China (*2*) is marked as group E. The nontyped strain from Japan isolated in 1998 is included in accession nos. LC202966 and LC202944 (*4*). We did not include the nontyped strain from South Africa, due to the lack of sequence information in GenBank. BTV strains were used as the outgroups. Number at each branch indicates a bootstrap value. Scale bars indicate nucleotide substitutions per site. BTV, bluetongue virus; EHDV, epizootic hemorrhagic disease virus.

The outer capsid protein VP2 of EHDV, encoded by Seg-2, is highly variable and is definitive for serotype determination (*2*). Seg-2/VP2 of YNDH/V079/2018 sharing sequence identities of 44.3%–50.9% nt and 31.0%–40.6% aa to previously recognized EHDV serotypes, which supports it as a distinct EHDV Seg-2 group (*2*), herein named group E (Figure, panel B). This finding coincides with the results of neutralization tests and indicates that YNDH/V079/2018 represents a novel serotype of EHDV.

We developed conventional RT-PCR and qRT-PCR targeting Seg-2 of YNDH/V079/2018 (Appendix Table 1) and used them in combination with cELISA and serum neutralization tests to trace progress of the infection in the sentinel animal (Appendix Table 2). We defined the earliest week in which the virus was detectable in the blood using qRT-PCR as infection week 1. We isolated YNDH/V079/2018 in week 2 from the sentinel calf, which experienced fever to 40.2°C, anorexia, and respiratory distress. Viral nucleic acid in the blood peaked in weeks 1–2; levels decreased gradually until none was detected at week 14. cELISA and neutralization antibodies first appeared at week 2, peaked in weeks 7–8, and remained elevated until week 17, when blood sampling ceased.

To determine the prevalence of YNDH/V079/2018, we tested 87 EDTA cattle blood samples from 3 farms in Mangshi County during April–October 2018 but detected no nucleic acid consistent with YNDH/V079/2018. Additional genome sequencing, type-specific diagnostic tests, and epidemiologic and pathogenic investigations of this novel EHDV are planned. Our study will help clarify the diversity of EHDV serotypes and the distribution and pathogenicity of this novel EHDV and its potential risk to ruminants.

Appendix Additional information about epizootic hemorrhagic disease virus, China.
