# Supplementary material for: Novel Serotype of Epizootic Hemorrhagic Disease Virus, China
Source: Emerg Infect Dis. 2020 Dec;26(12):3081–3. doi: 10.3201/eid2612.191301 (PMC7706924; doi:10.3201/eid2612.191301)
Supplement: Appendix — Additional information about epizootic hemorrhagic disease virus, China. [file 19-1301-Techapp-s1.pdf]

Article DOI: https://doi.org/10.3201/eid2612.191301

Novel Serotype of Epizootic Hemorrhagic Disease Virus, China

Appendix

Appendix Table 1. Primers and probe used for reverse transcription PCR (RT-PCR) and quantitative reverse transcription (RT-qPCR) of epizootic hemorrhagic disease virus targeting Segment 2 of the strain YNDH/V079/2018*

PCR type	Target gene	Probe and primer names	Primer sequence (5′-3′)	Nucleotide location	PCR products size, bp
RT-PCR	Seg-2	EHDV/V079-S2-F	GGCTCGGTTGCGTCTATTATG	1135–1156	999
		EHDV/V079-S2-R	TCCTTGAAGTCTCGGTAGTCG	2092–2113	
RT-qPCR	Seg-2	EHDV/V079-YG-S2-F	GCGCTCTAATTTGGCAGATAG	1093–1116	189
		EHDV/V079-YG-S2-R	AGCCGTTCCAAACCATAAGATAG	1033–1060	
		EHDV/V079-S2-Probe	FAM-TCGCAACCAGTCATCATCAAGATCGCT-BHQ1	950–971	

*The RT-qPCR reaction was conducted with One Step PrimeScript RT-PCR Kit (TaKaRa, https://www.takarabio.com) in a total volume of 20 μL containing 10 μL 2×One-Step RT-PCR Buffer III, 0.4 μL EX Taq HS DNA Polymerase (5 U/μL), 0.4 μL RT Enzyme Mix II, 0.4 μL primers (10 μM), 0.8 μL TaqMan Probe (10 μM), 4 μL RNA template, and RNase-free water to a final volume of 20 μL, which was performed in a 96-well plate using the ABI 7500 Real-Time PCR System (ABI, https://www.thermofisher.com) with the following thermal cycling conditions: 5 min at 42°C, 10 s at 95°C, and 40 cycles of 5 s at 95°C and 34 s at 60°C.

Appendix Table 2. Chronology of serologic and RT-qPCR assays of a calf infected with strain YNDH/V079/2018 of epizootic hemorrhagic disease virus, China, 2008*

Date	Week	Cycle threshold (C_t) value		C-ELISA inhibition (%)	Serum neutralization test titer
		Seg-9	Seg-2		
Aug 19	0	Negative	Negative	93.75	Negative
Aug 26	1	32.78	26.56	36.58	1:11
Sep 1	2	30.49	27.47	**27.63**	1:45
Sep 9	3	34.43	29.78	**21.06**	1:91
Sep 16	4	33.34	30.80	**15.62**	1:128
Sep 23	5	34.12	30.38	**11.43**	1:181
Sep 30	6	35.89	31.56	**10.02**	1:256
Oct 8	7	34.77	32.31	**8.36**	1:256
Oct 14	8	37.89	34.61	**10.14**	1:181
Oct 21	9	Negative	35.45	**9.62**	1:256
Oct 28	10	Negative	37.38	**10.62**	1:181
Nov 27	14	Negative	Negative	**11.57**	1:181
Dec 19	17	Negative	Negative	**12.73**	1:181

*RT-qPCR targeting Seg-9 of EHDV (*3*) and Seg-2 of YNDH/V079/2018 developed in this study. C_t values $\leq$38.0 for the Seg-2 RT-qPCR test are regarded as positive. Bold text indicate positive results using competitive ELISA test (ID Vet, https://www.id-vet.com) to detect antibodies against YNDH/V079/2018 in blood samples of the infected calf, according to the manufacturer's recommendations. Results are expressed as ratio of inhibition; samples were considered positive, if the percentage was <30%.

.

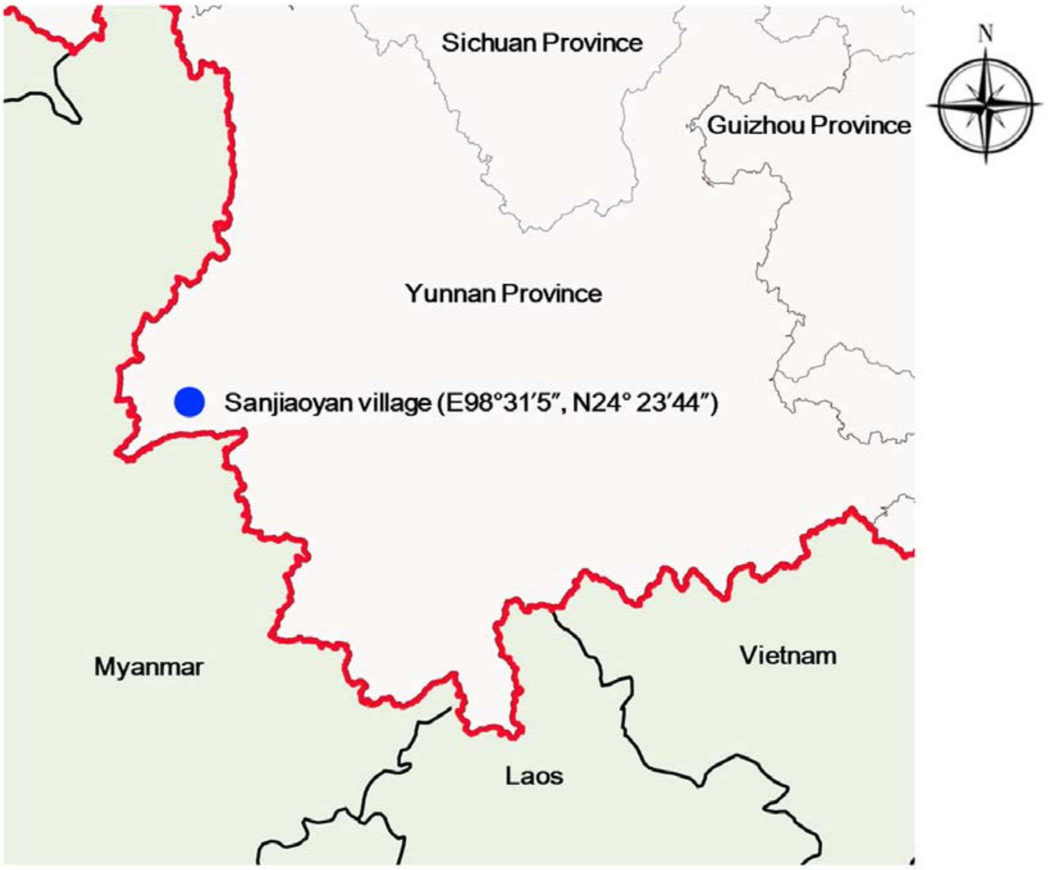

Appendix Figure 1. Geographic location of Sanjiaoyan village (blue dot, with longitude E98°31¢5^{2}, latitude N24°23¢44^{2} and altitude 880 m) located in Mangshi County, Dehong Prefecture, Yunnan Province of China. China’s boundaries with its neighbors are marked with solid red lines.

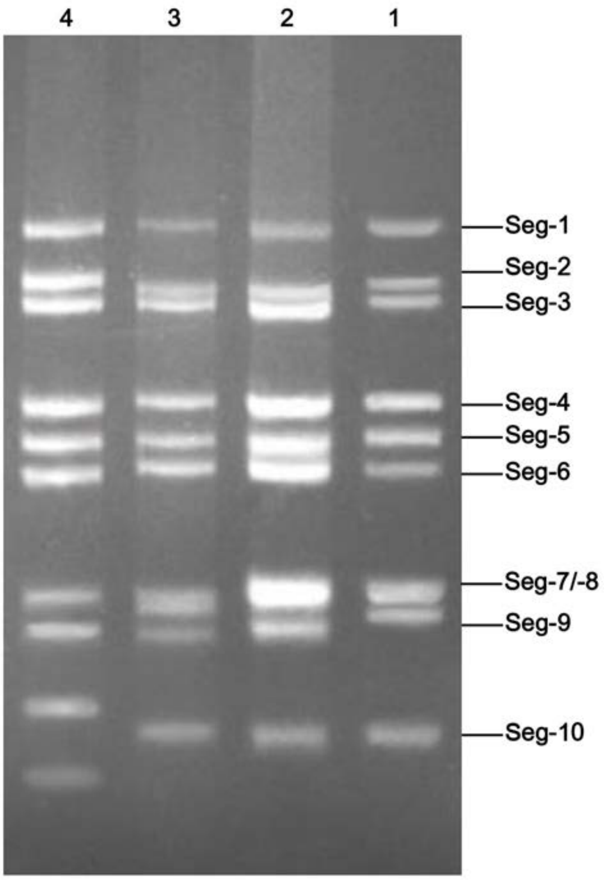

Appendix Figure 2. Agarose gel (2%) electrophoretic migration patterns of genomic double-stranded RNAs from *Orbivirus* species. Lane 1, YNDH/V079/2018; Lane 2, EHDV-5 (YNDH/V023/2014); Lane 3, bluetongue virus (BTV); Lane 4, chuzan virus (CHUV).

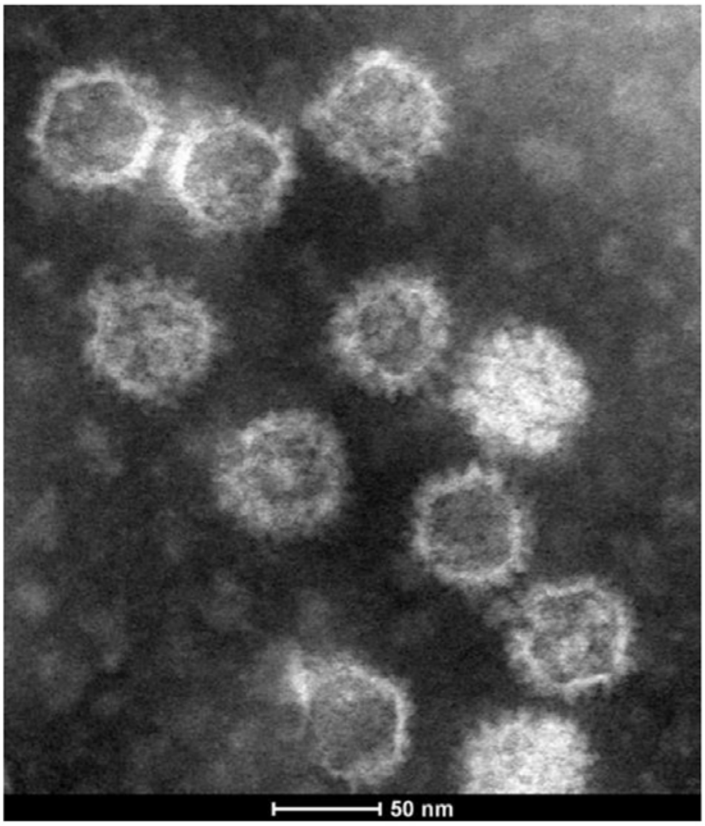

Appendix Figure 3. Electron micrographs of YNDH/V079/2018 particles with 2% potassium phosphotungstate negatively stained. Scale bar indicates 50 nm.
